# Effectiveness of non-pharmacological interventions for older adults with dementia: An umbrella review

**DOI:** 10.1016/j.ijnss.2026.06.008

**Published:** 2026-06-22

**Authors:** Taeko Saito, Natsumi Shimizu, Li Yao

**Affiliations:** a Faculty of Medical Care and Health Sciences Graduate School of Nursing, Nippon Medical School, Kanagawa, Japan; b Faculty of Nursing, Musashino University, Tokyo, Japan; c Graduate School of Nursing, Chiba University, Chiba, Japan

**Keywords:** Aged, Agitation, Anxiety, Complementary therapies, Dementia, Depression, Systematic review

## Abstract

**Objective:**

This umbrella review aimed to synthesize systematic reviews and meta-analyses to evaluate the effectiveness of non-pharmacological interventions (NPIs) on agitation, depression, anxiety, cognitive function, and quality of life in individuals living with dementia, and to examine the stability and methodological quality of the evidence base.

**Methods:**

Systematic searches of MEDLINE (via EBSCOhost), PubMed, CINAHL, PsycINFO, and Web of Science databases were conducted from inception to February 2025. Systematic reviews and meta-analyses evaluating NPIs for older adults with dementia were included. Methodological quality was assessed using A MeaSurement Tool to Assess Systematic Reviews 2 (AMSTAR 2). Evidence overlap was quantified using the corrected covered area (CCA), and effect estimates reported in the included reviews were synthesized narratively.

**Results:**

Twelve systematic reviews, including 147 randomized controlled trials, were included. Methodological quality varied across reviews, and all reviews had at least one non-critical weakness. Review overlap was very slight (CCA = 1.8 %). Across reviews, NPIs were associated with small but consistent reductions in agitation (standardized mean difference ([*SMD*] −0.25, 95 %*CI*: −0.36 to −0.13), depression ([*SMD*] −0.20, 95 %*CI*: −0.29 to −0.11), and anxiety ([*SMD*] −0.21, 95 %*CI*:−0.34 to −0.09). A modest improvement was observed in cognitive function ([*SMD*] 0.22, 95 %*CI*:0.11 to 0.34 ), whereas no significant effect was found for quality of life ([*SMD*] 0.08, 95 %*CI*: −0.15 to 0.32). The most consistent benefits were reported for information and communication technology-based interventions, massage and touch therapies, and physical exercise. Evidence certainty was moderate for agitation, depression, and anxiety, but low or very low for cognitive function and quality of life.

**Conclusion:**

NPIs were associated with small but consistent improvements in agitation, depression, anxiety, and cognitive function among older adults with dementia. Although effect sizes were modest and evidence certainty varied across outcomes, the findings support NPIs as key components of person-centered dementia care. Further high-quality research is needed to clarify optimal intervention modalities, dosage, mechanisms of action, and implementation strategies across diverse care settings.

## What is known?

• Behavioral and psychological symptoms of dementia (BPSD) are common and burdensome.
• Numerous systematic reviews have evaluated individual non-pharmacological interventions (NPIs), but findings are fragmented across modalities and settings.
• The comparative consistency and methodological quality of evidence across intervention types remain unclear.

## What is new?

• This umbrella review synthesizes 12 systematic reviews and meta-analyses and demonstrates small but consistent reductions in agitation and depression across multiple NPI modalities.
• Overlap across reviews was minimal (CCA 1.8 %), and evidence certainty was moderate for core BPSD outcomes.
• A cross-modal mechanistic framework is proposed to explain how sensory, activity-based, and technology-supported interventions may operate through overlapping physiological and psychosocial pathways.

## 1 Introduction

Dementia is a progressive neurocognitive disorder affecting approximately 57 million people worldwide, and its prevalence is projected to reach 139 million by 2050 [1,2]. In Japan, the proportion of older adults living with dementia is expected to reach approximately one in five, reflecting one of the most rapidly aging populations globally [1]. In response, Japan enacted the Dementia Basic Act in 2023, emphasizing person-centered care, early support, and social inclusion [3].

Beyond cognitive decline, up to 80 %–90 % of individuals with dementia experience behavioral and psychological symptoms of dementia (BPSD) during the disease course [4]. BPSD—including agitation, depression, anxiety, and psychosis—are strongly associated with caregiver burden, institutionalization, accelerated functional decline, and reduced quality of life [4,5]. Consequently, effective management of BPSD is central to dementia care across community, long-term care, and increasingly acute care settings. Although pharmacological treatments such as antipsychotics and antidepressants are commonly prescribed, their clinical benefits are modest and accompanied by substantial risks, including increased mortality, cerebrovascular events, sedation, and falls [[6], [7], [8]]. Accordingly, international clinical guidelines recommend non-pharmacological interventions (NPIs) as first-line strategies for the management of BPSD [9,10].

NPIs encompass a broad range of modalities, including music therapy, exercise, multisensory stimulation, light therapy, horticultural therapy, person-centered care approaches, and technology-based interventions such as information and communication technology (ICT) programs and socially assistive robots. Over the past decade, numerous systematic reviews and meta-analyses have evaluated specific NPI modalities. While many reports indicate modest improvements in agitation, depression, or cognitive function, findings remain fragmented across intervention types and care contexts [11,12]. Importantly, existing reviews differ substantially in methodological rigor, population characteristics, outcome definitions, and intervention “dose,” limiting cross-modal comparison and hindering mechanistic interpretation. Moreover, few syntheses integrate findings across diverse modalities to examine whether common physiological, psychosocial, or environmental regulatory mechanisms may underlie observed effects.

Given the proliferation of systematic reviews and meta-analyses on NPIs for dementia, a higher-level synthesis is warranted. Umbrella reviews provide a structured appraisal of review-level evidence, evaluate methodological quality, and identify consistency and gaps across interventions [13,14]. By integrating fragmented evidence across modalities and settings, an umbrella review can clarify the stability of NPI effects and provide a foundation for mechanism-informed and context-sensitive clinical application. Therefore, the aims of this umbrella review were to: 1) evaluate the effectiveness of diverse NPIs across major BPSD domains—specifically agitation, depression, and anxiety; and 2) synthesize evidence regarding secondary outcomes, including cognitive function and quality of life.

## 2 Methods

This study followed the Joanna Briggs Institute (JBI) methodology for umbrella reviews, which involves synthesizing evidence from existing systematic reviews and meta-analyses [14]. The study adhered to the Preferred Reporting Items for Systematic Reviews and Meta-Analyses (PRISMA) 2020 guidelines. The study protocol was prospectively registered in the PROSPERO database (CRD420250637728). Ethics approval was not required as all data were obtained from previously published studies.

### 2.1 Selection criteria and search strategy

The eligibility criteria were defined according to the Population, Intervention, Comparator, Outcomes, Type of study, Timing, and Setting (PICOTS) framework (Table 1). We included systematic reviews and meta-analyses focusing on adults (≥65 years) diagnosed with dementia of any subtype (e.g., Alzheimer’s disease, vascular dementia, Lewy body dementia, or mixed dementia) according to established diagnostic criteria (e.g., Diagnostic and Statistical Manual of Mental Disorders, International Classification of Diseases, or validated clinical assessments). Reviews primarily addressing mild cognitive impairment (MCI) without a formal dementia diagnosis, delirium without underlying dementia, or cognitive impairment secondary to medical conditions or treatments (e.g., chemotherapy-induced cognitive impairment, type 2 diabetes-related cognitive decline, or traumatic brain injury) were excluded. Studies that included mixed populations were eligible only if data for participants with dementia were reported separately or if participants with dementia constituted the majority of the sample.

**Table 1:** Inclusion explained by PICOTS framework.

Domain	Inclusion criteria	Exclusion criteria
Population (P)	Older adults diagnosed with dementia (any subtype) or mild cognitive impairment (MCI), residing in community, long-term care, assisted living, or hospital settings (including acute care).	Individuals without a formal diagnosis of dementia or MCI (e.g., cognitive impairment secondary to acute medical illness, traumatic brain injury, chemotherapy, or primary psychiatric disorders).
Intervention (I)	Non-pharmacological interventions (NPIs), including music therapy, physical exercise, massage/touch therapy, multisensory stimulation, light therapy, horticultural therapy, person-centered care approaches, information and communication technology (ICT)-based programs, and socially assistive robots.	Pharmacological interventions as primary treatment; combined interventions where the independent effect of NPIs could not be determined.
Comparator (C)	Usual care, standard care, waitlist control, minimal intervention, or alternative NPIs.	Studies without a comparator group included within the review (e.g., uncontrolled before–after studies only).
Outcomes (O)	Primary outcomes: Behavioral and psychological symptoms of dementia (BPSD), including agitation, depression, and anxiety. Secondary outcomes: Cognitive function and quality of life (QOL).	Reviews not reporting quantitative patient-level outcomes related to BPSD, cognition, or QOL; reviews focusing solely on caregiver outcomes.
Timing (T)	No restriction on duration of follow-up; outcomes measured at post-intervention and/or follow-up as defined by review authors.	Reviews not reporting timing of outcome assessment.
Setting (S)	Community, long-term care facilities, assisted living environments, and hospital settings (including acute care).	Animal studies; exclusively laboratory-based experimental studies without clinical populations.
Study design	Systematic reviews and meta-analyses of randomized controlled trials (RCTs) or controlled clinical trials.	Narrative reviews, scoping reviews, conference abstracts without full text, grey literature, and reviews lacking systematic methodology.

To comprehensively identify published systematic reviews of NPIs, database searches were conducted across the following databases from their inception through February 2025: MEDLINE (via EBSCOhost), PubMed, CINAHL, PsycINFO, and Web of Science. The search terms included “dementia,” “non-pharmacological intervention,” “agitation,” “BPSD,” “aged,” and related subject headings. A search strategy was developed in consultation with a professional librarian. The reference lists of eligible reviews were also screened. Subject headings, keywords, and free-text terms were combined using the Boolean operators “AND” and “OR,” tailored to each database. Searches were conducted across multiple databases, and detailed search strategies for all databases are provided in Appendix A.

### 2.2 Study selection

All retrieved records were imported into Rayyan, and duplicate entries were removed before screening. Two reviewers (T. Saito and N. Shimizu) independently screened the titles and abstracts according to the predefined eligibility criteria. Potentially eligible full-text articles were independently assessed by two reviewers (T. Saito and N. Shimizu). Any discrepancies were resolved through discussion and, when necessary, consultation with a third reviewer (Y. Li) to reach a consensus. The study selection process was documented using a PRISMA 2020 flowchart [15]. Studies were excluded if their titles or abstracts indicated the inclusion of participants without dementia or MCI or if the interventions included both non-pharmacological and pharmacological therapies. Full-text articles were excluded if they were meta-analyses with insufficient data or where meta-analyses had not been performed in terms of the primary outcome of this study.

### 2.3 Data extraction

Data were independently extracted by two reviewers (T. Saito and N. Shimizu) using the JBI Data Extraction Form for Review of Systematic Reviews and Research Syntheses. This data extraction form aligned with the predefined eligibility criteria and the PROSPERO protocol. The form was piloted on three reviews and refined before full data extraction. Discrepancies were resolved by discussion and consensus. The extracted data included review characteristics (first author, year of publication, and country), review objectives, participant and setting characteristics, number and design of the included primary studies, total sample size, and funding sources. Detailed information on NPIs was collected, including intervention categories, modality, duration, intensity, and comparator conditions (e.g., usual care or pharmacological treatment). Outcome data included effects on three predefined core BPSD domains: agitation, depression, and anxiety, as well as secondary outcomes (cognitive function and quality of life). The extracted information included outcome measurement instruments, effect size metrics (standardized mean difference [*SMD*], mean difference [*MD*], and 95 %*CI*s), statistical models, heterogeneity estimates (*I*^2^), and the authors’ conclusions and reported limitations. When multiple reviews included overlapping primary studies, all eligible reviews were retained to provide a comprehensive overview of the evidence. In cases of duplicate datasets within meta-analyses, the most complete dataset was prioritized for quantitative synthesis. The review authors were contacted for clarification or additional data when necessary.

### 2.4 Methodological quality appraisal

The methodological quality of the included systematic reviews and meta-analyses was independently assessed by two reviewers (T. Saito and N. Shimizu) using A Measurement Tool to Assess Systematic Reviews, version 2 (AMSTAR 2) [16]. AMSTAR 2 is a 16-item instrument designed to evaluate the methodological quality of systematic reviews of randomized and non-randomized studies. In accordance with AMSTAR 2 guidelines, critical domains were defined a priori, including protocol registration, comprehensiveness of the literature search, justification of excluded studies, adequacy of risk-of-bias assessment, appropriateness of statistical methods, consideration of risk-of-bias in the interpretation of results, and assessment of publication bias. Each item was rated as “Yes,” “Partial Yes,” “No,” or “Unclear.” Consistent with AMSTAR 2 recommendations, no overall numerical scores were calculated. Instead, overall confidence in each review’s results was categorized as high, moderate, low, or critically low, based on the presence of critical flaws and non-critical weaknesses. Any discrepancies between reviewers were resolved through discussion and consensus. If necessary, a third reviewer (Y. Li) was consulted.

### 2.5 Overlap assessment

To evaluate the degree of overlap among the included systematic reviews, we calculated the corrected covered area (CCA) using the method proposed by Pieper et al. [17]. The CCA quantifies the extent to which primary studies are shared across multiple reviews and is calculated as follows:

CCA = (Nr − Ns)/[Ns × (*r* − 1)](1) where *N*s represents the number of unique primary studies, Nr is the total number of primary study occurrences across all reviews, and *r* is the number of reviews included. The degree of overlap was interpreted according to established thresholds: 0–5 % (very slight), 6 %–10 % (slight), 11 %–15 % (moderate), and >15 % (high).

### 2.6 Data synthesis

Study characteristics were summarized in tables and synthesized narratively in accordance with the JBI methodology for umbrella reviews. Given the heterogeneity of intervention modalities, outcome measures, and reporting formats across the included systematic reviews, quantitative pooling was not used as the primary analytical strategy. Instead, this umbrella review relied primarily on the effect estimates and narrative summaries reported in the included reviews. To avoid misinterpretation as de novo meta-analysis, summary estimates reported in this review were derived from effect sizes presented in the included systematic reviews and are provided for interpretative purposes only. No re-analysis of primary study data was conducted, and all effect estimates were derived from the included reviews to avoid double-counting and preserve the integrity of the original meta-analyses.

### 2.7 Primary approach

The primary synthesis focused on three core predefined BPSD outcomes (agitation, depression, and anxiety). For each outcome, the findings were synthesized by examining the magnitude, direction, and consistency of the reported effects across the reviews. Broader secondary outcomes (cognitive function and quality of life) were summarized descriptively when quantitative comparisons were not feasible.

### 2.8 Exploratory secondary analyses

When the numerical data were sufficiently comparable across the reviews, limited exploratory analyses were conducted to enhance interpretability. When MDs were reported without SMDs, they were converted to SMDs using established methods (e.g., dividing the MD by the pooled standard deviation reported in the review or estimated from available summary statistics, such as interquartile ranges or ranges). This allowed us to compare effect estimates across different outcome scales. Rather than conducting de novo meta-analyses of primary studies, we summarized the effect directions and ranges of the SMDs across the reviews.

### 2.9 Summarizing effects across non-pharmacological intervention modalities

To improve interpretability, subgroup summaries were generated by grouping reviews according to the primary NPI modality investigated (e.g., ICT-based interventions, physical activity, music therapy, and light therapy). For each subgroup, we assessed the consistency and direction of the effects (e.g., consistent positive effects, mixed findings, or no clear effect). We reported the available range of effect sizes. Due to the limited number of reviews contributing to the data within each subgroup and methodological heterogeneity across the reviews, formal meta-regression analyses or statistical interaction tests were not performed. No imputation of missing data was performed. Reviews with incomplete numerical reporting were included only in the narrative synthesis.

### 2.10 Protocol deviations

Minor deviations from the registered PROSPERO protocol occurred due to variability in outcome reporting across the included reviews. Although the protocol specified broader psychological and functional outcomes, a quantitative synthesis was feasible only for agitation, depression, and anxiety. These deviations were transparently documented and did not alter the overall direction or interpretation of the findings.

## 3 Results

### 3.1 Study characteristics

**Fig. 1:**
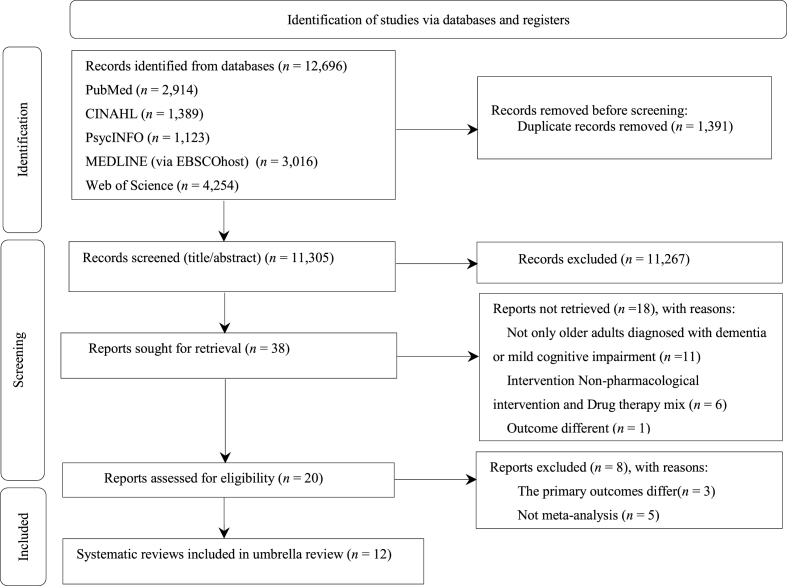
The PRISMA flow diagram of the study selection process.

Fig. 1 presents a PRISMA flow diagram of the study selection process. A total of 12 systematic reviews were included in this umbrella review [[18], [19], [20], [21], [22], [23], [24], [25], [26], [27], [28], [29]]. All the included reviews synthesized randomized controlled trials, totaling 147 primary studies. The included reviews covered a broad range of NPIs, including ICT-based interventions (e.g., socially assistive robots), music-based interventions, physical exercise and mind-body therapies, light therapy, horticultural therapy, massage and touch interventions, and person-centered care approaches. Most reviews focused on long-term care facilities and community-based settings. Notably, none of the included reviews specifically targeted the acute care setting. The most frequently reported outcomes were agitation, depression, anxiety, cognitive function, and quality of life. Agitation was commonly measured using the Cohen-Mansfield Agitation Inventory (CMAI). At the same time, depression and anxiety were typically assessed using validated scales such as the Geriatric Depression Scale (GDS), Cornell Scale for Depression in Dementia (CSDD), and Rating Anxiety in Dementia (RAID). Cognitive function was often measured using the Mini-Mental State Examination (MMSE), and quality of life was commonly evaluated using instruments such as the Quality of Life in Alzheimer’s Disease (QoL-AD) scale. The detailed characteristics of the included reviews are presented in Appendix B.

### 3.2 Methodological quality

The AMSTAR-2 assessment indicated that all included reviews had at least one non-critical weakness. Common methodological limitations included a lack of protocol registration, incomplete justification for excluded studies, and limited assessment of publication bias. Overall, four reviews were rated as high-quality and two as moderate-quality. However, several reviews were judged to be of low or critically low quality, primarily because of the absence of a priori protocols, incomplete reporting of excluded studies, and insufficient consideration of risk of bias in the interpretation of the findings. Accordingly, the findings of this umbrella review should be interpreted with caution, particularly when concluding reviews of lower methodological quality. A detailed summary of the AMSTAR 2 assessment is presented in Appendix C.

### 3.3 Overlap assessment

Across the 12 systematic reviews, 147 unique primary studies were identified. The total number of primary studies across the reviews (Nr) was 176. The CCA was calculated as follows: CCA = (176 − 147)/[147 × (12 − 1)](2), which showed a CCA of 1.8 %, indicating a very slight overlap among the included reviews. A detailed overlap matrix is provided in Appendix D.

### 3.4 Clinical outcomes

These summary estimates were derived from representative effect sizes reported in the included meta-analyses and are presented for interpretive purposes only, not as results of a new quantitative synthesis. Forest plots illustrating the summary effect estimates reported in the included reviews are presented in Appendix E. Additionally, Supplementary material Appendix F provides a detailed mapping of NPI effectiveness across clinical outcomes by intervention modality.

#### 3.4.1 Agitation

Ten reviews reported outcomes related to agitation. Across NPIs, effect estimates reported across the included reviews consistently indicated a small but consistent reduction in agitation, with effect estimates approximately [*SMD*]−0.25 (95 % *CI* −0.36 to −0.13; *I*^2^ = 58 %), indicating generally consistent findings despite moderate heterogeneity. Interventions demonstrating notable benefits included ICT-based interventions (e.g., socially assistive robots such as Psychological Attachment Robot [PARO]), massage, and touch therapy, which showed the largest individual effects, and participatory horticultural therapy, which consistently reduced agitation Appendix E (A).

#### 3.4.2 Depression

Nine reviews assessed depressive symptoms. Effect estimates across the included reviews indicated a small but consistent reduction in depression, with summary estimates around [*SMD*] −0.20 (95 %*CI* −0.29 to −0.11; *I*^2^ = 0). Modalities demonstrating positive effects included ICT-based interventions, physical exercise, horticultural therapy, and music-based interventions. In contrast, light and mind-body therapies consistently showed no significant effects on depression across the reviews Appendix E (B).

#### 3.4.3 Anxiety

Four reviews examined anxiety outcomes. The effect estimates across the included reviews indicated a small but consistent reduction across reviews in anxiety, with summary estimates around [*SMD*] −0.21 (95 %*CI* −0.34 to −0.09; *I*^2^ = 0). The absence of heterogeneity suggested consistent findings. Although some ICT-based interventions were reported as showing relatively large improvements in individual reviews, the overall effect remained modest. Given the limited number of contributing studies and participants, further high-quality research is needed to strengthen the evidence on anxiety reduction Appendix E (C).

#### 3.4.4 Cognitive function

Seven reviews investigated cognitive outcomes. Effect estimates across the included reviews indicated a small but statistically significant improvement in cognitive function, with summary estimates around [*SMD*] 0.22 (95 %*CI* 0.11 to 0.34; *I*^2^ = 0). Interventions contributing to cognitive improvement included horticultural therapy, physical activity and mind-body interventions, phototherapy, and person-centered care programs, several of which showed statistically significant positive effects in individual reviews. In contrast, bright-light therapy and pet robot interventions did not demonstrate statistically significant effects, as their CIs crossed zero. Overall, although the summary estimate was small, the consistent direction of the effects across the reviews suggests that certain NPIs may contribute to modest improvements in cognitive outcomes Appendix E (D).

#### 3.4.5 Quality of life

Three reviews assessed quality-of-life outcomes. The pooled analysis indicated no statistically significant improvement in quality of life following NPI interventions ([*SMD*] = 0.08, 95 %*CI*: −0.15 to 0.32; *I*^2^ = 0). Individual reviews evaluating pet robot interventions, person-centered care, and music therapy reported small positive effects; however, the confidence intervals crossed zero in all cases, indicating no statistical significance. These findings suggest that while certain NPIs may provide subjective benefits, the current evidence does not demonstrate a consistent improvement in overall quality of life among people with dementia Appendix E (E).

#### 3.4.6 Summary across the intervention modalities

Across the intervention modalities, ICT-based interventions demonstrated the most consistent evidence for reducing agitation and depression, with small to large effects depending on the intervention type [[19], [21], [26]]. Massage and touch therapies were consistently associated with small reductions in agitation [28]. Physical activity and mind–body approaches showed small improvements in agitation and cognitive function, while their effects on depression and anxiety were variable [[22], [24]]. Photo therapy and horticultural therapy demonstrated large improvements in cognitive function [[20], [25]], whereas light therapy produced mixed findings, with generally small or negligible effects on agitation and depression [[18], [23]]. Music therapy was associated with small improvements in agitation and depression but negligible effects on anxiety, cognitive function, and quality of life [29]. Person-centered care interventions showed small improvements in agitation and cognitive function but negligible effects on quality of life [27]. A detailed mapping of intervention modalities and clinical outcomes is provided in Appendix F.

### 3.5 Subgroup analyses by intervention modality

Subgroup summaries were conducted by primary NPI modality. ICT-based interventions demonstrated the most consistent beneficial effects on agitation and depression outcomes. Massage and touch therapies showed relatively larger reductions in agitation than the other modalities. Physical exercise and mind-body therapies demonstrated modest but consistent improvements in cognitive outcomes. In contrast, music and horticultural therapies showed more variable effects across different outcomes, and light therapy yielded mixed findings, particularly for depressive symptoms. Formal statistical interaction testing was not performed due to the limited number of reviews per subgroup.

### 3.6 Certainty of evidence

Downgrading was primarily due to methodological limitations identified through AMSTAR-2 assessments and to imprecision related to small sample sizes in several contributing reviews. Where certainty ratings were reported, these were extracted directly from the included reviews rather than reassessed in this umbrella review.

### 3.7 Sensitivity analysis

Sensitivity analyses were conducted to examine the influence of reviews rated as low or critically low quality according to AMSTAR 2. Excluding these reviews did not change the overall direction of effects across reviews for agitation or depression. However, the effect estimates for anxiety and quality of life became more uncertain owing to the reduced sample size and wider *CI*s.

These findings suggest that while the overall conclusions remained stable for the core BPSD outcomes, caution is warranted when interpreting the results for secondary outcomes.

## 4 Discussion

### 4.1 Summary of findings

This umbrella review synthesized evidence from 12 systematic reviews and meta-analyses comprising 147 randomized controlled trials and evaluated the effectiveness of NPIs for BPSD. Across reviews, NPIs were associated with small but consistent reductions in agitation, depression, and anxiety, together with modest improvements in cognitive function. Although the magnitude of the effects was modest, the consistency of findings across intervention modalities suggests that NPIs can contribute meaningfully to dementia care. In contrast, evidence regarding quality of life was inconsistent and did not demonstrate a statistically significant overall effect. The certainty of evidence varied across outcomes because several included reviews had methodological limitations identified by the AMSTAR 2 assessment.

### 4.2 Cross-modal interpretation of non-pharmacological interventions’ effects

Although the included interventions differed substantially in content, several common patterns emerged. Interventions involving sensory stimulation, physical activity, social engagement, and environmental modification were consistently associated with small improvements in agitation, anxiety, or depressive symptoms. These findings suggest that NPIs may exert their effects through multiple interacting pathways, including physiological regulation, emotional engagement, and adaptation of the care environment. Rather than producing large symptom reductions [30], NPIs may contribute to symptom stabilization and improved adaptation to dementia-related challenges. The convergence of beneficial effects across diverse intervention modalities supports the view that no single intervention is universally effective and that individualized approaches are likely required.

### 4.3 Contextual moderators

The effectiveness of NPIs appeared to be influenced by both care setting and participant characteristics. Most evidence originated from community and long-term care settings, whereas evidence from acute hospital settings was limited. This imbalance restricts generalizability to more medically complex environments. In addition, intervention responsiveness may vary according to dementia severity, baseline symptom burden, and individual abilities [31]. These findings suggest that NPIs are most effective when tailored to individuals' needs and capacities and implemented within supportive care environments.

### 4.4 Strengths and limitations

This umbrella review has several strengths. It synthesized evidence across major NPI modalities, incorporated methodological quality assessment using AMSTAR 2, and evaluated overlap among reviews. The low corrected covered area suggested minimal duplication of primary studies across included reviews, strengthening confidence in the overall findings. Several limitations should also be acknowledged. First, the review relied on review-level data and therefore could not examine participant-level moderators or intervention fidelity in detail. Second, substantial heterogeneity in interventions and outcome measures limited direct comparison across studies. Third, the available evidence was predominantly derived from community and long-term care settings, restricting generalizability to acute hospital environments [31]. Finally, although statistical heterogeneity was generally low across several outcomes, conceptual heterogeneity remained considerable due to differences in participant characteristics, intervention delivery, and clinical contexts.

## 5 Conclusion

This umbrella review found that non-pharmacological interventions were associated with small but consistent improvements in agitation, depression, anxiety, and cognitive function among older adults with dementia. Although the magnitude of effects was modest, these findings support current recommendations that NPIs should be considered first-line approaches for dementia care. Future research should focus on mechanism-informed, context-sensitive interventions and evaluate their implementation across diverse care settings, particularly acute care hospitals.

## Data availability statement

The datasets generated during and/or analyzed during the current study are available from the corresponding author upon reasonable request.

## CRediT authorship contribution statement

**Taeko Saito:** Conceptualization, Methodology, Formal analysis, Writing – original draft, Writing – review & editing. **Natsumi Shimizu:** Methodology, Data curation, Formal analysis. **Li Yao:** Formal analysis.

## Funding

This study was supported by a research grant from the Insurance Union of Societies Related to Nursing. The article processing charge (APC) was supported by the DAIGAKU TOKUBETSU KENKYUHI Grant (Musashino University).

## Declaration of competing interest

The authors have declared no conflict of interest. During the preparation of this work, the author(s) used ChatGPT to assist with English translation. After using this tool, the author(s) reviewed and edited the content as needed and take full responsibility for the content of the published article.
