# Supplementary material for: Effectiveness of non-pharmacological interventions for older adults with dementia: An umbrella review
Source: Int J Nurs Sci. 2026 Jun 22;13(4):380–6. doi: 10.1016/j.ijnss.2026.06.008 (PMC13424413; doi:10.1016/j.ijnss.2026.06.008)
Supplement: Multimedia component 1 [file mmc1.docx]

**非药物干预对老年痴呆患者的有效性：一项伞式综述**

Taeko SAITO, Natsumi SHIMIZU, Li Yao

【**摘要**】

**目的** 系统性整合相关综述和meta分析，评估非药物干预（non-pharmacological interventions, NPIs）在老年痴呆患者中对躁动、抑郁、焦虑、认知功能及生活质量的影响，并评估证据基础的稳定性与方法学质量。

**方法** 检索MEDLINE、PubMed、CINAHL、PsycINFO和Web of Science数据库从建库至2025年2月的文献。采用系统评价测量评估工具（第 2 版）评估符合条件的系统综述和meta分析。效应估计值通过标准化均差进行汇总，而各综述之间的重叠部分则使用校正覆盖面积进行量化。

**结果** 共纳入了12项系统综述，包含147项随机对照试验。各综述的方法学质量存在差异，综述间的重叠度极低（CCA = 1.8%）。采用NPIs后老年痴呆患者轻度但持续的躁动(*SMD*=−0.25，95%*CI*: −0.36至−0.13)，抑郁(*SMD*=−0.20, 95% *CI*: −0.29至−0.11)，以及焦虑水平均降低(SMD=−0.21, 95% *CI*：−0.34至−0.09)；认知功能方面观察到适度改善(SMD=0.22，95% *CI*：0.11至0.34)，而生活质量则未发现显著影响(SMD=0.08，95% *CI*：−0.15至0.32)。信息与通信技术干预、按摩与触摸疗法以及体育锻炼显示出有益效果。关于躁动、抑郁和焦虑的证据确定性为中等，而认知功能和生活质量的证据确定性较低或极低。

**结论** 非药物干预与痴呆老年人在躁动、抑郁、焦虑和认知功能方面的轻微但持续改善相关。尽管各结果的效果量较小，且证据的确定性存在差异，但研究结果支持将非药物干预措施作为以个体为中心的痴呆护理的关键组成部分。未来需要开展更多高质量的研究，以明确不同护理环境中最佳的干预方式、剂量、作用机制及实施策略。

【**关键词**】老年人；躁动；焦虑；痴呆；抑郁；补充疗法；系统综述

通信作者：Taeko Saito, E-mail:[taeko-saito@nms.ac.jp](mailto:taeko-saito@nms.ac.jp)
