# Supplementary material for: Effectiveness of non-pharmacological interventions for older adults with dementia: An umbrella review
Source: Int J Nurs Sci. 2026 Jun 22;13(4):380–6. doi: 10.1016/j.ijnss.2026.06.008 (PMC13424413; doi:10.1016/j.ijnss.2026.06.008)
Supplement: Multimedia component 2 [file mmc2.docx]

Appendix A. Detailed search strategy for databases

| Search ID | Search terms | Results |
| --- | --- | --- |
| Database: PubMed Search date: February 25, 2025 | | |
| #1 | "dementia"[MeSH Terms] OR "cognitive dysfunction"[MeSH Terms] OR "cognition disorders"[MeSH Terms] OR "memory disorders"[MeSH Terms] | 331,732 |
| #2 | "dement*"[Title/Abstract] OR "cognit* dysfunct*"[Title/Abstract] OR "cognit* disorder*"[Title/Abstract] OR "cognit* disturb*"[Title/Abstract] OR "cognit* impair*"[Title/Abstract] OR "cognit* defici*"[Title/Abstract] OR "cognit* declin*"[Title/Abstract] OR "memory disorder*"[Title/Abstract] | 300,396 |
| #3 | #1 OR #2 | 459,333 |
| #4 | "aged"[MeSH Terms] OR "aged, 80 and over"[MeSH Terms] | 3647,549 |
| #5 | "elder*"[Title/Abstract] OR "presenile"[Title/Abstract] OR "geriatric*"[Title/Abstract] OR "elderly patient*"[Title/Abstract] OR "older adult*"[Title/Abstract] OR "older patient*"[Title/Abstract] | 552,016 |
| #6 | #4 OR #5 | 3,820,443 |
| #7 | "non-pharmacological"[Title/Abstract] OR "nonpharmacological"[Title/Abstract] OR "non-drug"[Title/Abstract] | 25,847 |
| #8 | "Animal Assisted Therapy"[MeSH Terms] OR "aromatherapy"[MeSH Terms] OR "Art Therapy"[MeSH Terms] OR "Cognitive Behavioral Therapy"[MeSH Terms] OR "Cognitive Training"[MeSH Terms] OR "Exercise Therapy"[MeSH Terms] OR "Horticultural Therapy"[MeSH Terms] OR "Mind-Body Therapies"[MeSH Terms] OR "Music Therapy"[MeSH Terms] OR "Occupational Therapy"[MeSH Terms] OR "phototherapy"[MeSH Terms] OR "psychotherapy"[MeSH Terms] OR "Recreation Therapy"[MeSH Terms] | 379,286 |
| #9 | "behavioral intervention"[Title/Abstract] OR "Cognitive Rehabilitation"[Title/Abstract] OR "cognitive stimulation"[Title/Abstract] OR "Life Review"[Title/Abstract] OR "massage"[Title/Abstract] OR "Reality orientation"[Title/Abstract] OR "Reminiscence Therapy"[Title/Abstract] OR "Reminiscence"[Title/Abstract] | 24,824 |
| #10 | #7 OR #8 OR #9 | 399,005 |
| #11 | "Randomized Controlled Trial"[Publication Type] OR "Controlled Clinical Trial"[Publication Type] OR "Comparative Study"[Publication Type] | 2,491,134 |
| #12 | "Controlled Clinical Trials as Topic"[MeSH Terms] OR "Randomized Controlled Trials as Topic"[MeSH Terms] | 188,831 |
| #13 | "random*"[Title/Abstract] OR "control*"[Title/Abstract] OR "compar*"[Title/Abstract] | 10,797,425 |
| #14 | #11 OR #12 OR #13 | 11,648,769 |
| #15 | "#14 AND #10 AND #6 AND #3 | 3,291 |
| #16 | Article Language: English, Japanese; Age: 80 and over: 80+ years; Aged:65+years | 2,914 |
| Database: CINAHL Complete (EBSCOhost) Search date: February 25, 2025 | | |
| #1 | (MH "Dementia") OR (MH "Cognition Disorders") OR (MH "Memory Disorders") OR AB dement* OR AB "Cognit* Dysfunct*" OR AB "Cognit* Disorder*" OR AB "Cognit* Disturb*" OR AB "Cognit* impair*" OR AB "Cognit* defici*" OR AB "Cognit* declin*" OR AB "Memory disorder*" | 127,801 |
| #2 | (MH "Aged") OR AB elder* OR AB presenile OR AB geriatric* OR AB "elderly patient*" OR AB "older adult*" OR AB "older patient*" | 993,666 |
| #3 | AB "non-pharmacological" OR AB nonpharmacological OR AB "non-drug" | 8,612 |
| #4 | (MH "Pet Therapy") OR (MH "Aromatherapy") OR (MH "Art Therapy") OR AB "behavioral intervention" OR (MH "Cognitive Therapy") OR AB "Cognitive Rehabilitation" OR AB "cognitive stimulation" OR AB "Cognitive Training" OR (MH "Activity and Exercise Enhancement (Iowa NIC)") OR AB "Horticultural Therapy" OR AB "Life Review" OR AB massage | 41,053 |
| #5 | AB "Mind-Body Therapies" OR (MH "Music Therapy") OR (MH "Occupational Therapy") OR (MH "Phototherapy") OR (MH "Psychotherapy") OR AB "Reality orientation" OR (MH "Recreation Therapy (Iowa NIC)") OR AB "Reminiscence Therapy" OR AB Reminiscence | 61,016 |
| #6 | #3 OR #4 OR #5 | 105,860 |
| #7 | #1 AND #2 AND #6 | 2,869 |
| #8 | (MH "Randomized Controlled Trials") OR AB random* OR AB control* OR AB compar* | 1,682,537 |
| #9 | #7 AND #8 | 1,389 |
| Database: PsycINFO (EBSCOhost) Search date: February 25, 2025 | | |
| #1 | DE "Dementia" OR DE "Cognitive Impairment" OR AB dement* OR AB "Cognit* Dysfunct*" OR AB "Cognit* Disorder*" OR AB "Cognit* Disturb*" OR AB "Cognit* impair*" OR AB "Cognit* defici*" OR AB "Cognit* declin*" OR AB "Memory disorder*" | 158,573 |
| #2 | DE "Aged" OR AB elder* OR AB presenile OR AB geriatric* OR AB "elderly patient*" OR AB "older adult*" OR AB "older patient*" | 323,129 |
| #3 | AB "non-pharmacological" OR AB nonpharmacological OR AB "non-drug" | 7,098 |
| #4 | DE "Aromatherapy" OR AB "behavioral intervention" OR AB "Cognitive Rehabilitation" OR AB "cognitive stimulation" OR AB "Cognitive Training" OR DE "Horticulture Therapy" OR AB "Life Review" OR AB massage OR AB "Mind-Body Therapies" OR DE "Music Therapy" OR DE "Occupational Therapy" OR DE "Psychotherapy" | 107,382 |
| #5 | AB "Reality orientation" OR DE "Recreation Therapy" OR AB "Reminiscence Therapy" OR AB "Reminiscence" | 3,026 |
| #6 | #3 OR #4 OR #5 | 116,128 |
| #7 | (DE "Randomized Controlled Trials") OR AB random* OR AB control* OR AB compar* | 1,609,826 |
| #8 | #1 AND #2 AND #6 AND #7 | 1,123 |
| Database: MEDLINE (EBSCOhost) Search date: February 25, 2025 | | |
| #1 | MH "Memory Disorders+" OR MH "Cognition Disorders+" OR MH "Cognitive Dysfunction+" OR MH "Dementia+" OR related title/abstract terms | 453,271 |
| #2 | MH "Aged+" OR elder* OR presenile OR geriatric* OR "elderly patient*" OR "older adult*" OR "older patient*" | 3,798,473 |
| #3 | non-pharmacological interventions including Animal Assisted Therapy, Aromatherapy, Art Therapy, Cognitive Behavioral Therapy, Cognitive Rehabilitation, Cognitive Training, Exercise Therapy, Horticultural Therapy, Music Therapy, Occupational Therapy, Phototherapy, Psychotherapy, Recreation Therapy, Reminiscence Therapy and related terms | 419,251 |
| #4 | MH "Randomized Controlled Trials as Topic+" OR MH "Controlled Clinical Trials as Topic+" OR random* OR control* OR compar* | 10,758,653 |
| #5 | #1 AND #2 AND #3 AND #4 | 3,475 |
| #6 | Limits applied: English or Japanese language; Aged ≥65 years; peer-reviewed publications | 3,016 |
| Database: Web of Science Core Collection Search date: February 25, 2025 | | |
| #1 | TS=(Dementia OR Cognitive Dysfunction OR Cognition Disorders OR Memory Disorders OR dement* OR related cognitive impairment terms) | 492,791 |
| #2 | TS=(Aged OR "Aged, 80 years and over" OR elder* OR presenile OR geriatric* OR "elderly patient*" OR "older adult*" OR "older patient*") | 4,983,252 |
| #3 | TS=("non-pharmacological" OR nonpharmacological OR "non-drug" OR Animal Assisted Therapy OR Aromatherapy OR Art Therapy OR Cognitive Behavioral Therapy OR Cognitive Rehabilitation OR Cognitive Training OR Exercise Therapy OR Horticultural Therapy OR Music Therapy OR Occupational Therapy OR Phototherapy OR Psychotherapy OR Recreation Therapy OR Reminiscence Therapy OR related terms) | 224,951 |
| #4 | TS=("Randomized Controlled Trial" OR "Controlled Clinical Trial" OR Comparative Study OR random* OR control* OR compar*) | 19,623,025 |
| #5 | #1 AND #2 AND #3 AND #4 | 4,417 |
| #6 | Limits applied: English language | 4,254 |

Appendix B. Characteristics of the included studies

| Author(s) | Title | Year | Objective | Number of studies/years/participants | Primary summary | Scales |
| --- | --- | --- | --- | --- | --- | --- |
| Fong, et al.[23] | The effects of light therapy on sleep, agitation and depression in people with dementia: a systematic review and meta-analysis of randomized controlled trials | 2023 | To evaluate the effects of light therapy on sleep disturbances, agitation, and depression in people with dementia. | 11 studies (2003–2021), *n* = 648. | Light therapy significantly reduced nighttime awakenings (*SMD* = 0.31; *P* = 0.02). No significant effect was found for wake after sleep onset or depression. | Agitation: CMAI Depression: CSDD, NPI |
| Cho , et al. [21] | The effectiveness of non-pharmacological interventions using information and communication technologies for behavioral and psychological symptoms of dementia: a systematic review and meta-analysis | 2022 | To examine the effectiveness of ICT-based non-pharmacological interventions for BPSD and identify moderators of intervention effects. | 16 studies (2015–2021); sample size not reported. | Significant moderate effect on overall BPSD (*SMD* = −0.664). Large effect on depression (*SMD* = −1.088) and moderate effect on agitation (*SMD* = −0.586). No significant effect on anxiety or apathy. | Agitation: CMAI, CBOS, BARS Anxiety: RAD Depression: CSDD, GDS |
| Rashid, et al.[26] | The effectiveness of a therapeutic robot, ‘Paro’, on behavioural and psychological symptoms, medication use, total sleep time and sociability in older adults with dementia: a systematic review and meta-analysis | 2023 | To evaluate the effectiveness of the therapeutic robot Paro on BPSD, medication use, sleep, and sociability. | 12 studies (2013–2020), *n* = 1,461. | Paro had moderate effects on medication use and small effects on anxiety, agitation, and depression. No meaningful effect on sleep time. | Agitation: BARS, K-CMAI, CMAI-SF Anxiety: RAID Depression: CSDD, GDS |
| Liu, et al.[28] | Effect of massage and touch on agitation in dementia: a meta-analysis | 2025 | To assess the effects of massage and touch on agitation and determine optimal intervention design. | 17 studies (2002–2022), *n* = 980. | Massage and touch significantly reduced agitation; short-term interventions (≤ 4 weeks) were more effective. Greater effects were seen in individuals with less severe dementia. | Agitation: CMAI |
| Saragih,et al. [27] | A meta-analysis of person-centered care interventions for improving health outcomes in persons living with dementia | 2024 | To evaluate the efficacy of person-centered care (PCC) in improving outcomes in people with dementia. | 17 studies (2004–2019), *n* = 3,073. | PCC improved cognitive function, but had no significant effects on ADLs, agitation, depression, or QOL. | Agitation: BARS, CMAI, PAS Depression: CSDD, GDS Cognition: MARS, MMSE ADLs: BI, MBI QOL: DQoL, QUALID, QoL-AD |
| van der Steen, et al.[29] | Music-based therapeutic interventions for people with dementia (review) | 2025 | To evaluate the effect of active music interventions on cognitive and neuropsychiatric outcomes. | 30 studies (1993–2022), *n* = 1,720. | Music therapy significantly reduced depression and some behavioral problems. No significant effects on agitation, QOL, or cognition. | Depression: GDS, CSDD, MADRS, AD-RD Agitation: CMAI, NPI subscale Anxiety: RAID, STAI-A, HAMD QOL: QoL-AD, CBS-QoL, ADRQL Cognition: MMSE, SIB |
| Lu, et al.[25] | Phototherapy improves cognitive function in dementia: A systematic review and meta-analysis | 2023 | To assess the effectiveness of phototherapy on cognitive function. | 12 studies (2001–2022), *n* = 766. | Phototherapy improved cognitive function and reduced nighttime awakenings, but had no significant effect on agitation or depression. | Cognition: MMSE Depression: CSDD Agitation: CMAI |
| Kouloutbani,et al. [24] | Physical exercise as a nonpharmacological intervention for the treatment of neuropsychiatric symptoms in persons with dementia | 2023 | To evaluate the effect of physical exercise on neuropsychiatric symptoms in dementia. | 13 studies (2011–2018), *n* = 1,925. | (Not provided in original table; may need completion.) | Depression: BDI, HDRS, GDS, MADRS Agitation: Agitated Behavior Scale |
| Wang, et al. [22] | Mind–body therapies for older adults with dementia: a systematic review and meta‐analysis | 2022 | To evaluate the effects of mind–body therapies (MBTs) in dementia. | 9 studies (2011–2019), *n* = 583. | Tai Chi significantly improved cognitive function; yoga and aromatherapy showed potential benefits for depression and QOL. | Depression: CSDD, GDS Agitation: PAS, RASS Anxiety: HADS, RAID QOL: ICECAP-O, QoL-AD |
| Zhao, et al.[20] | Effectiveness of horticultural therapy in people with dementia: a quantitative systematic review | 2020 | To evaluate the effectiveness of horticultural therapy on cognition, agitation, positive emotion, and engagement. | 14 studies (2002–2018), *n* = 411. | Participatory horticultural therapy significantly improved cognition, agitation, positive emotion, and engagement. Ornamental horticulture showed no significant effects. | Cognition: MMSE, TSI, HDS-R Agitation: CMAI, BARS, ABS Positive Emotion: modified DCM, OERS |
| Leng, et al.[19] | Pet robot intervention for people with dementia: a systematic review and meta-analysis of randomized controlled trials | 2019 | To evaluate the efficacy of pet robot interventions for BPSD, cognitive function, and QOL. | 8 studies (2013–2018), *n* = 502. | Pet robot interventions significantly reduced agitation, depression, and anxiety/apathy. Effects on cognition and QOL were unclear. | Agitation: CMAI-SF, BARS Depression: CSDD, GDS Cognition: MMSE, ACE, GDS QOL: QoL-AD, QUALID |
| Forbes, et al.[18] | Light therapy for improving cognition, activities of daily living, sleep, challenging behaviour, and psychiatric disturbances in dementia (review) | 2014 | To examine the effectiveness of light therapy on cognition, ADLs, sleep, challenging behaviors, and psychiatric symptoms. | 11 studies (1998–2011), *n* = 499. | Light therapy improved nighttime awakenings, but showed no significant effects on agitation, depression, sleep efficiency, or cognition. | Cognition: MMSE Sleep: Wrist actigraphy Agitation: CMAI, BEHAVE-AD, NPI-NH Depression: NPI, CSDD |

*Note*: BPSD = behavioral and psychological symptoms of dementia. CMAI = Cohen-Mansfield Agitation Inventory. CSDD = Cornell Scale for Depression in Dementia. NPI = Neuropsychiatric Inventory. CBOS = Cohen-Mansfield Behavioral Observation Scale. BARS = Brief Agitation Rating Scale. RAD = Rating Anxiety in Dementia. GDS = Geriatric Depression Scale.CMAI-SF = Cohen-Mansfield Agitation Inventory – Short Form. K-CMAI = Korean Version of Cohen-Mansfield Agitation Inventory. RAID = Rating Anxiety in Dementia. PAS = Pittsburgh Agitation Scale. MMSE = Mini-Mental State Examination. MARS = Memory Assessment Rating Scale. BI = Barthel Index. MBI = Modified Barthel Index. DQoL = Dementia Quality of Life Instrument. QUALID = Quality of Life in Late-Stage Dementia Scale. QoL-AD = Quality of Life – Alzheimer’s Disease. MADRS = Montgomery–Åsberg Depression Rating Scale. AD-RD = Alzheimer’s Disease Related Depression Scale. CMAI = Cohen-Mansfield Agitation Inventory. NPI subscale = Neuropsychiatric Inventory subscale (Agitation/Aggression subscale). STAI-A = State-Trait Anxiety Inventory – State Anxiety Scale. HAMD = Hamilton Rating Scale for Depression. CBS-QoL = Caregiver Burden Scale-Quality of Life. ADRQL = Alzheimer’s Disease Related Quality of Life. SIB = Severe Impairment Battery. NPI = Neuropsychiatric Inventory. NPI-NH = Neuropsychiatric Inventory-Nursing Home version. BEHAVE-AD = Behavioral Pathology in Alzheimer’s Disease Rating Scale.

Appendix C. A complete summary of the AMSTAR 2 assessment

| Systematic review | C1 | C2 | C3 | C4 | C5 | C6 | C7 | C8 | C9 | C10 | C11 | C12 | C13 | C14 | C15 | C16 |
| --- | --- | --- | --- | --- | --- | --- | --- | --- | --- | --- | --- | --- | --- | --- | --- | --- |
| Forbes, 2014 [18] | Y | P | Y | Y | Y | Y | Y | Y | Y | N | Y | Y | Y | Y | Y | Y |
| Leng, 2019 [19] | Y | P | Y | N | Y | Y | P | P | P | N | Y | Y | Y | Y | N | Y |
| Zhao, 2020 [20] | Y | P | Y | P | Y | Y | P | Y | P | N | Y | Y | Y | Y | Y | Y |
| Cho, 2022 [21] | Y | Y | Y | P | Y | Y | Y | P | Y | Y | Y | Y | Y | Y | Y | Y |
| Wang, 2022 [22] | Y | P | Y | N | Y | Y | N | N | P | N | Y | Y | Y | Y | Y | Y |
| Fong, 2023 [23] | Y | N | Y | Y | Y | Y | P | P | P | Y | Y | N | Y | Y | N | Y |
| Kouloutbani, 2023 [24] | Y | Y | Y | P | Y | Y | N | P | P | N | Y | Y | Y | Y | Y | Y |
| Lu, 2023 [25] | Y | P | Y | P | Y | Y | P | Y | P | N | Y | Y | Y | Y | Y | Y |
| Rashid, 2023 [26] | Y | Y | Y | Y | Y | Y | P | P | Y | Y | Y | Y | Y | Y | Y | Y |
| Saragih, 2024 [27] | Y | Y | Y | Y | Y | Y | Y | Y | Y | Y | Y | Y | Y | Y | Y | Y |
| Liu, 2025 [28] | Y | Y | Y | Y | Y | Y | Y | Y | Y | Y | Y | N | Y | Y | Y | Y |
| van der Steen, 2025 [29] | Y | Y | Y | Y | Y | Y | Y | Y | Y | Y | Y | Y | Y | Y | Y | N |

*Note*：Y=Yes, P=Partially Yes, N=No. This table summarizes the overall confidence ratings derived from the AMSTAR 2 assessment. Four reviews were rated as high confidence, two as moderate confidence, four as low confidence, and two as critically low confidence. AMSTAR 2 items: C1 (research questions and inclusion criteria), C2 (protocol registration), C3 (justification for study designs), C4 (comprehensive search strategy), C5 (study selection in duplicate), C6 (data extraction in duplicate), C7 (excluded studies list), C8 (description of included studies), C9 (risk of bias assessment), C10 (funding sources of included studies), C11 (statistical synthesis), C12 (impact of risk of bias on meta-analysis), C13 (risk of bias considered in interpretation), C14 (investigation of heterogeneity), C15 (publication bias), and C16 (conflicts of interest).

| Primary study | Forbes,2014 | Leng,2019 | Zhao, 2020 | Cho,  2022 | Wang,2022 | Fong,2023 | Kouloutbani,2023 | Liu,  2023 | Rashid,2023 | Saragih,2023 | Lu,  2023 | van der Steen,  2025 |
| --- | --- | --- | --- | --- | --- | --- | --- | --- | --- | --- | --- | --- |
| Reference (No) | 18 | 19 | 20 | 21 | 22 | 23 | 24 | 25 | 26 | 27 | 28 | 29 |
| Dowling et al. (2007) |  |  |  |  |  | 1 |  |  |  |  | 1 |  |
| Riemersma-vander Lek et al. (2008) | 1 |  |  |  |  | 1 |  |  |  |  |  |  |
| Burns et al. (2009) | 1 |  |  |  |  | 1 |  |  |  |  | 1 |  |
| McCurry et al. (2011) | 1 |  |  |  |  | 1 |  |  |  |  | 1 |  |
| Onega et al. (2016) |  |  |  |  |  | 1 |  |  |  |  | 1 |  |
| Hjetland et al. (2021) |  |  |  |  |  | 1 |  |  |  |  | 1 |  |
| Kolberg et al. (2021) |  |  |  |  |  | 1 |  |  |  |  | 1 |  |
| Moyle et al. (2017) |  | 1 |  | 1 |  |  |  |  | 1 |  |  |  |
| Moyle et al. (2013) |  | 1 |  |  |  |  |  |  | 1 |  |  |  |
| Petersen et al. (2017) |  | 1 |  |  |  |  |  |  | 1 |  |  |  |
| Raglio et al. (2015) |  |  |  |  |  |  |  |  |  | 1 |  | 1 |
| Nowak (2008) | 1 |  |  |  |  |  |  |  |  |  | 1 |  |
| Graf et al. (2001) | 1 |  |  |  |  |  |  |  |  |  | 1 |  |

Appendix D. Matrix of overlapping primary studies among systematic reviews

**Appendix B. Characteristics of the included studies. *(Continued)***

*Note*: The value “1” indicates that the corresponding primary study was included once in the respective systematic review. Blank cells indicate that the study was not included. This matrix was used to assess the overlap of primary studies across the included systematic reviews.

Appendix E. The effects of non-pharmacological interventions on (A) agitation (B) depressive symptoms, (C)Anxiety (D) Cognitive Function(E) Quality of Life in older adults with dementia*（Continued）*

Appendix E. The effects of non-pharmacological interventions on (A) agitation (B) depressive symptoms, (C)Anxiety (D) Cognitive Function(E) Quality of Life in older adults with dementia. *Note*: ICT = information and communication technology. NPIs = non-pharmacological interventions.

Appendix F. A detailed mapping of the outcomes by intervention modality is provided

| Non-Pharmacological Group | Non-Pharmacological Content | Study | Agitation | Interpretation | Depression | Interpretation | Anxiety | Interpretation | Cognitive function | Interpretation | Quality of life | Interpretation |
| --- | --- | --- | --- | --- | --- | --- | --- | --- | --- | --- | --- | --- |
| ICT-based interventions | Therapeutic robot “PARO” | Rashid, 2023 [26] | ✔︎ | Small effect | ✔︎ | Small effect | ✔︎ | Negligible |  |  |  |  |
|  | ICT interventions | Cho, 2022 [21] | ✔︎ | Small effect | ✔︎ | Large | ✔︎ | Moderate |  |  |  |  |
|  | Pet robot | Leng, 2019 [19] | ✔︎ | Small effect | ✔︎ | Negligible | ✔︎ | Small effect | ✔︎ | Negligible | ✔︎ | Negligible |
| Physical activity/ mind–body approaches | Mind–body therapies | Wang, 2022 [22] | ✔︎* |  | ✔︎ | Moderate | ✔︎* |  | ✔︎ | Small effect | ✔︎* |  |
|  | Physical exercise | Kouloutbani,  2023 [24] |  |  | ✔︎ | Negligible |  |  |  |  |  |  |
|  | Massage and touch | Liu, 2025 [28] | ✔︎ | Small effect |  |  |  |  |  |  |  |  |
| Photo therapy | Photo therapy | Lu, 2023 [25] | ✔︎ | negligible | ✔︎ | Negligible |  |  | ✔︎ | Large |  |  |
| Light therapy | Light therapy | Fong, 2023 [23] | ✔︎ | Small effect | ✔︎ | Small effect |  |  |  |  |  |  |
|  | Bright light therapy | Forbes, 2014 [18] | ✔︎ | Negligible | ✔︎ | Negligible |  |  | ✔︎ | Large |  |  |
| Horticultural therapy | Horticultural therapy | Zhao, 2020 [20] | ✔︎ | Negligible |  |  |  |  | ✔︎ | Large |  |  |
| Multiple/  complex interventions | Person-centered care | Saragih, 2024 [27] | ✔︎ | Small effect | ✔︎ |  |  |  | ✔︎ | Small effect | ✔︎ | Negligible |
| Music therapy | Music | van der Steen, 2025 [29] | ✔︎ | Small effect | ✔︎ | Small effect | ✔︎ | Negligible | ✔︎ | Negligible | ✔︎ | Negligible |

*Note*: NPIs = non-pharmacological interventions. * Effect estimates were derived from narrative synthesis because a meta-analysis was not performed.
